# Achilles tendon enthesitis evaluated by MRI assessments in patients with axial spondyloarthritis and psoriatic arthritis: a report of the methodology of the ACHILLES trial

**DOI:** 10.1186/s12891-020-03775-4

**Published:** 2020-11-21

**Authors:** Xenofon Baraliakos, Philipp Sewerin, Eugenio de Miguel, Effie Pournara, Christine Kleinmond, Annette Wiedon, Frank Behrens

**Affiliations:** 1 Rheumazentrum Ruhrgebiet Herne, Ruhr-University Bochum, 44649 Herne, Germany; 2 Department and Hiller Research Unit of Rheumatology, University Hospital Düsseldorf, Düsseldorf, Germany; 3 Department of Rheumatology, University Hospital La Paz, Madrid, Spain; 4 Novartis Pharma AG, Basel, Switzerland; 5 ClinProject GmbH, Eurasburg, Germany; 6 Novartis Pharma GmbH, Nürnberg, Germany; 7 CIRI/Rheumatology and Fraunhofer TMP, Goethe-University, Frankfurt, Germany

**Keywords:** Achilles tendon, Enthesitis, Spondyloarthritis, PsAMRIS, Bone erosion, Bone oedema, MRI

## Abstract

**Background:**

The currently available scoring methods for enthesitis are often measures of pain but not of inflammation at entheseal sites. The Outcome Measures in Rheumatology Clinical Trials (OMERACT) psoriatic arthritis (PsA) magnetic resonance imaging (MRI) scoring system (PsAMRIS) assesses inflammation and damage in PsA and was particularly developed for the hands. The ACHILLES trial used clinical measures for heel enthesitis in combination with MRI scoring based on PsAMRIS.

**Methods:**

Patients (age ≥ 18 years) with spondyloarthritis (SpA) and PsA were included in the trial if they presented with clinical and MRI-positive heel enthesitis. MRI of the affected heel was performed at three time points: screening, Week 24 and Week 52. Inflammatory MRI findings (tendinitis, bursitis and bone marrow oedema [BME]) in the area of the Achilles tendon and/or plantar aponeurosis, periarticular inflammation of the ankle joint and heel erosion were assessed qualitatively (absent/present). In addition, BME and bone erosion were quantitatively assessed based on PsAMRIS, where their proportion was compared to the volume of the affected bone. Mean scores of BME and bone erosion quantification were calculated, and the mean composite score (based on PsAMRIS) was calculated based on the individual score of each subject for periarticular inflammation, BME and bone erosion and further extended for bursitis and tendinitis. Modifications to PsAMRIS were introduced by categorising oedema length as ≤/> 0.5 cm and locating bone erosion.

**Conclusions:**

In ACHILLES, MRI was used to assess and evaluate heel enthesitis. Due to the lack of a validated scoring system for heel enthesitis at the time of ACHILLES initiation, this trial applied quantitative scoring based on PsAMRIS, with specific adaptations for the heel.

**Trial registration:**

National Clinical Trial Registry, NCT02771210. Registered 13 May 2016.

**Supplementary Information:**

The online version contains supplementary material available at 10.1186/s12891-020-03775-4.

## Background

Enthesitis is a key feature of axial spondyloarthritis (axSpA) and psoriatic arthritis (PsA), and pain at entheseal sites is a key clinical sign [1]. Enthesitis can occur at many sites in patients with SpA, but the heel (Achilles and plantar fascia insertions) is affected most frequently [2, 3].

Although enthesitis is usually clinically assessed by scores like Leeds Enthesitis Index (LEI), Maastricht Ankylosing Spondylitis Enthesitis Score (MASES) or Spondyloarthritis Research Consortium of Canada (SPARCC), these scores are often a measure of pain rather than a true measure of inflammation at the entheseal sites [4].

Magnetic resonance imaging (MRI) represents a sensitive tool to recognise enthesitis in both bone and soft tissues [5], especially in cases of a potential disconnect between pain at entheseal sites and objective signs of inflammation. The international Outcome Measures in Rheumatology Clinical Trials (OMERACT) MRI in arthritis working group developed the OMERACT PsA MRI scoring system (PsAMRIS) to evaluate inflammation and damage in PsA of the hands [5, 6]. The pathological features included in the PsAMRIS for peripheral PsA were synovitis, tenosynovitis, periarticular inflammation, bone oedema, bone erosion and bone proliferation [6]. Despite the high prevalence of heel enthesitis, there was no MRI scoring method available that addresses the morphologic peculiarities of the foot. Due to the lack of a validated scoring system, Yanaba et al. adapted the PsAMRIS for the heel. They divided the foot into four different regions (forefoot, midfoot, hindfoot and ankle) and applied individual PsAMRIS for each area and used PsAMRIS to analyse foot MRIs [7].

ACHILLES (NCT02771210) focuses on heel enthesitis in SpA, investigating clinical and imaging heel enthesitis endpoints. In the absence of a validated MRI scoring system for the heel at the time of study initiation, the ACHILLES trial used PsAMRIS with adaptations for heel enthesitis to assess MR images.

The current manuscript describes the methodology used to score heel enthesitis of patients with PsA and axial SpA enrolled in the ACHILLES study.

## Methods

### Study design

ACHILLES is a two-treatment-arm, randomised, parallel-group, double-blind, placebo-controlled study in patients with PsA and axial SpA (Additional file 1). Patients were randomised in a ratio of 1:1 to receive either secukinumab or matching placebo at baseline, Weeks 1, 2, 3 and 4 and once every 4 weeks thereafter. Patients on placebo switched to active secukinumab treatment starting at Week 24.

### Patients

A total of 204 patients (age ≥ 18 years; 102 in the secukinumab 150 or 300 mg group and 102 in the placebo group) with active SpA (peripheral or axial) were included in the ACHILLES trial if they presented with clinical and MRI-positive heel enthesitis: clinical heel enthesitis defined as swelling and tenderness at the insertional site of the Achilles tendon into the calcaneus (binary pain assessment present/absent), and MRI-positive heel enthesitis defined as tendinitis with/without bursitis and/or bone marrow oedema (BME) with/without concomitant erosions in the insertional area of the Achilles tendon and/or the plantar aponeurosis. MRI-positive heel enthesitis was interpreted by either the local radiologist or rheumatologist at the individual study site.

### Image acquisition

MRI of the affected foot/ankle was performed by imaging technicians on standard MRI systems with magnetic field strengths of at least 1.5 Tesla and with the technical capability of performing foot MRI examinations. The MRI study protocol was standardized for all participating centres in an imaging manual and consisted of two mandatory sequences (T1-weighted turbo spin-echo/fast spin-echo in sagittal and transversal orientation and short inversion time inversion-recovery [STIR] in sagittal and transversal orientation) (Additional file 2).

MRIs were performed at three time points: screening, Week 24 and Week 52. No preparative drugs, contrast agents or radionuclide agents were used during the procedure.

### Assessments of heel MRI characteristics

Heel MRI was assessed both qualitatively and quantitatively. The qualitative assessment of heel MRI characteristics (absent/present) was performed as follows (Table 1): (i) tendinitis in the Achilles tendon and/or plantar aponeurosis, (ii) tendinitis in the Achilles tendon and/or plantar aponeurosis: insertional area, (iii) bursitis in the area of the Achilles tendon and/or plantar aponeurosis, (iv) BME in the area of the Achilles tendon and/or plantar aponeurosis, (v) periarticular inflammation of the ankle joint and (vi) bone erosion.

**Table 1:** Description of qualitative MRI parameters assessed by the central readers (absent/present)

Parameter Name	Parameter Description
Tendinitis	Tendinitis in the Achilles tendon and/or plantar aponeurosis
Tendinitis: insertional area	Tendinitis in the area of Achilles tendon insertion and/or hyperintense signal in the plantar aponeurosis at the site of insertion at the calcaneus
Bursitis	Bursitis in the area of the Achilles tendon and/or plantar aponeurosis
Bone oedema	BME in the insertion of the Achilles tendon in the upper part of the calcaneus and/or in the insertion of the plantar aponeurosis in the lower part of the calcaneus
Periarticular inflammation	Periarticular inflammation of the ankle joint
Bone erosion	Bone erosion in the calcaneus

*MRI* Magnetic resonance imaging

The quantitative assessment of heel MRI characteristics (based on PsAMRIS) is as follows (Table 2): (i) bone oedema; proportion of bone oedema was compared to the volume of the affected bone: 0 = absent, 1 = 1–33% of bone oedematous, 2 = 34–66% of bone oedematous, 3 = ≥ 67% of bone oedematous and (ii) bone erosion; proportion of bone erosion was compared to the volume of the affected bone: 0 = absent, 1 = 1–10% of bone eroded, 2 = 11–20% of bone eroded, …, 10 = 91–100% of bone eroded.

**Table 2:** Quantitative assessment of MRI findings as graded by the central readers

Parameter Name	Value Range
Bone oedema, PsAMRIS	0 = absent 1 = 1–33% 2 = 34–66% 3 = ≥ 67%
Bone oedema, adapted to heel	0 = absent 1a = 1–33% with oedema length ≤ 0.5 cm 1b = 1–33% with oedema length > 0.5 cm 2 = 34–66% 3 = ≥ 67%
Bone erosion, PsAMRIS	0 = absent 1 = 1–10% 2 = 11–20% 3 = 21–30% 10 = 91–100%

*MRI* Magnetic resonance imaging, *PsAMRIS* Outcome Measures in Rheumatology psoriatic arthritis MRI scoring system

The mean scores of heel MRI characteristics such as bone oedema quantification and bone erosion quantification will be calculated. The mean score of bone oedema quantification will be calculated based on the individual scores of each subject (0 = absent, 1 = 1–33%, 2 = 34–66%, 3 = ≥ 67%). Similarly, the mean score of bone erosion quantification will be calculated based on the individual scores of each subject (0 = absent, 1 = 1–10%, 2 = 11–20%, …, 10 = 91–100%).

The mean composite score based on PsAMRIS will be calculated based on the individual scores of each subject for periarticular inflammation, bone oedema and bone erosion as follows: periarticular inflammation (0 = absent, 1 = present), bone oedema (0 = absent, 1 = 1–33%, 2 = 34–66%, 3 = ≥ 67%) and bone erosion (0 = absent, 1 = 1–10%, 2 = 11–20%, …, 10 = 91–100%). Similarly, the mean composite score based on PsAMRIS, extended for bursitis and tendinitis, will be calculated based on the individual scores of each subject for the following parameters: periarticular inflammation (0 = absent, 1 = present), bone oedema (0 = absent, 1 = 1–33%, 2 = 34–66%, 3 = ≥ 67%), bone erosion (0 = absent, 1 = 1–10%, 2 = 11–20%, …, 10 = 91–100%), bursitis (0 = absent, 1 = present) and tendinitis (0 = absent, 1 = present).

Active inflammation was defined as ‘yes’ if at least one of the following parameters was present: tendinitis (Achilles tendon/plantar aponeurosis insertion), bursitis (Achilles tendon/plantar aponeurosis), bone oedema (Achilles tendon/plantar aponeurosis), periarticular inflammation and bone oedema (PsAMRIS). Active inflammation will be determined automatically by the reading software (Table 3) after completion of the manual read process. In case one or more read parameters cannot be determined for a data set (i.e. one image), the readers have the option to mark the parameters as ‘N/A’ (not applicable) and comment on the reasons for their decision.

**Table 3:** Parameters determined automatically by the reading software

Parameter Name	Parameter Description	Value Range
Active inflammation	Signs of an active inflammation	Yes / no / N/A
Value	Dependencies	
	List of direct parameters • Tendinitis (Achilles tendon) • Bursitis (Achilles tendon) • Bone oedema (Achilles tendon) • Tendinitis (plantar aponeurosis) • Bursitis (plantar aponeurosis) • Bone oedema (plantar aponeurosis) • Periarticular inflammation (PsAMRIS) • Quantification of bone oedema (PsAMRIS)	
Yes	In case at least one of the direct parameters above is ‘yes’	
No	In case all of the direct parameters above are ‘no’	
N/A	This value can only be set manually by consensus read when at least one of the direct parameters above is ‘N/A’, and none are ‘yes’	

*N/A* Not applicable, *PsAMRIS* Outcome Measures in Rheumatology psoriatic arthritis MRI scoring system

### Adaptations to the heel

Since PsAMRIS was developed initially for the hands, additional parameters were introduced to allow for a more accurate representation of the heel in terms of quantifying bone oedema and locating bone erosion. Bone oedema with 1–33% of bone oedematous was further specified by oedema length ≤/> 0.5 cm.

Bone erosions were further specified, if categorised as at least one and not more than 10, by assessing the localisation as follows: bone erosion in the area of the Achilles tendon, bone erosion in the area of the plantar aponeurosis and bone erosion in the area ‘Other’.

**Fig. 1:**
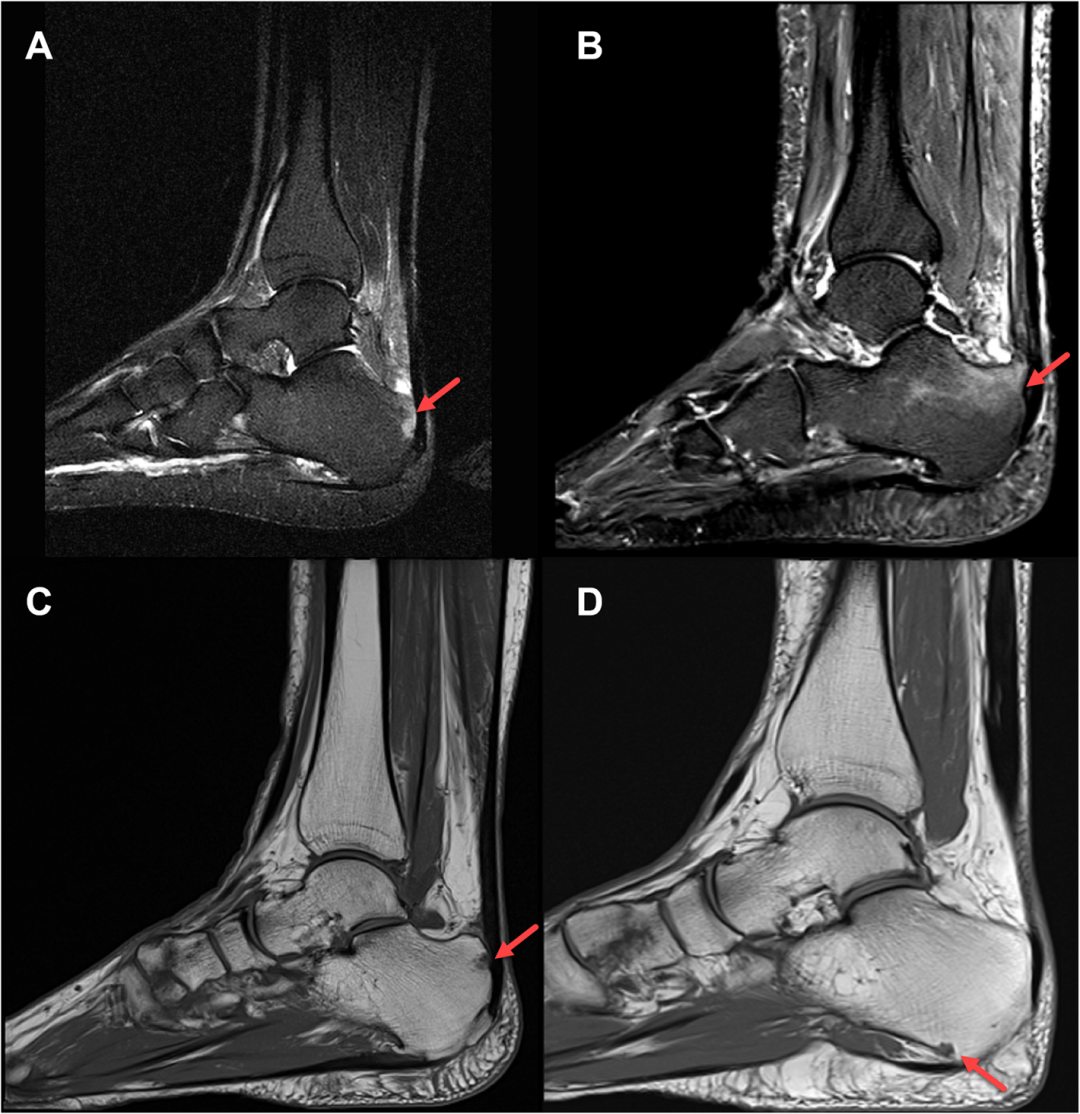
MRIs of bone oedema and bone erosion. **a** Quantification of bone oedema; 1–33% of bone being oedematous with an oedema length ≤ 0.5 cm. **b** Quantification of bone oedema; 1–33% of bone being oedematous with an oedema length > 0.5 cm. **c** Location of bone erosion in the area of the Achilles tendon. **d** Location of bone erosion in the area of the plantar aponeurosis. MRI, magnetic resonance imaging

MRIs of bone oedema and bone erosion of patients with active SpA are presented in Fig. 1; heel-specific adaptations are indicated with an arrow.

### Central reading scheme

The central read process was implemented in a consensus-read fashion with two readers, blinded for the identification of study centre, patient, treatment or visit/date of scan. The conditions for a consensus read depended on the reading scores: (i) > 1 deviation in the PsAMRIS scoring of the parameters ‘Quantification of Bone Oedema’ and ‘Quantification of Bone Erosion’ and (ii) discrepancy in any of the qualitative reading parameters. In case of a deviation in scoring for ‘Quantification of Bone Oedema’ and ‘Quantification of Bone Erosion’ of 1, the average value was recorded.

To start the read process, a reading task was assigned to the readers on the reading system. One reading task was defined as the image data of all available MRI time points of one patient. The MRIs of one reading task were reviewed according to the scoring forms for each time point. Each reading task was organised in a randomised independent temporal presentation, i.e., images regarding MRI time points were presented to the readers in a blinded fashion.

**Fig. 2:**
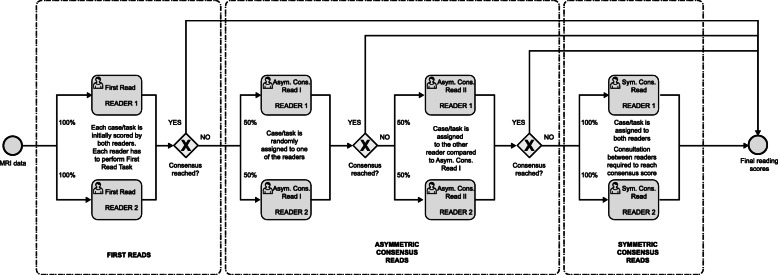
Consensus reading scheme. Asym., asymmetric; Cons., consensus; MRI, magnetic resonance imaging; Sym., symmetric

The detailed reading scheme is presented in Fig. 2. A brief reading scheme is as follows: (i) First Reads: Each reading task was initially rated by each reader, (ii) Asymmetric Consensus Reads: If no consensus was achieved in the First Reads, the case was randomly assigned to one of the readers. This reader now had the opportunity to change his own scores, while previous scores of both readers were displayed. If no consensus scoring was achieved after this step, the case was assigned to the other reader who then had the opportunity to change his scores, while previous scores of both readers were displayed and (iii) Symmetric Consensus Reads: If no consensus was achieved, the case was assigned to both readers simultaneously, and they have to agree to a consensus score by consultation to finish the read.

The clinical information of individual patients was not available to the readers in the read process, and there was no time limit for the completion of a single read.

## Discussion

The evaluation of enthesitis in SpA is challenging, either with clinical or with imaging approaches. The currently available clinical indices to score enthesitis in axial and peripheral SpA (LEI, MASES and SPARCC) have certain limitations with varying specificity, validity and reliability [8]. Although the number of entheseal sites differs in each of these indices, physical examinations of the entheseal sites to assess pain and tenderness are applied and recorded as binary enthesitis scores (1 for ‘present’ and 0 for ‘absent’) [8]; the outcome of the physical examination largely depends on the intensity of the pressure applied and the individual pain perception of the patient. In terms of the specificity of identifying tenderness in entheseal sites, the outcome might be confounded for fibromyalgia and/or mechanical stress as many entheseal points lie close to the joints and they also serve as tender points in fibromyalgia [9]. In consideration of the existing differences in and limitations of the discussed clinical indices, imaging techniques might be more sensitive but also specific to assess enthesitis.

ACHILLES is the largest study so far investigating both clinical and imaging endpoints with blinded, centrally read MRI data on enthesitis. MRIs were evaluated qualitatively and quantitatively for morphologic parameters representing inflammatory heel enthesitis; improvement or worsening of heel enthesitis on an MRI was assessed at three different time points for up to 1 year. In ACHILLES, the quantification of bone oedema and bone erosion was performed according to PsAMRIS as no validated scoring method for heel enthesitis in PsA and SpA was available at the time of study set-up.

To take into account the different morphology of the heel compared to the hand, for example, the calcaneus is much bigger than the carpal bones, some adaptations of the PsAMRIS have been introduced to the score. Two modifications were introduced to quantify bone oedema and to locate bone erosion. To quantify bone oedema, two sub-categories were introduced based on oedema length ≤ 0.5 cm and > 0.5 cm, and the location of bone erosion was assessed either in the Achilles tendon area or in the plantar aponeurosis area. Bone erosions typically develop in areas of compression and were reported earlier in the proximal portion of the Achilles tendon area in patients with SpA [10], emphasising the importance of locating erosions in areas such as the Achilles tendon in patients with SpA.

The calculation of mean scores allows a better description of the quantification of bone oedema/bone erosion for the overall population and also allows discrimination between different treatment groups; the scores used in ACHILLES have been chosen based on PsAMRIS. Composite scores take into account different pathologies that may characterise heel enthesitis; two composite scores were used in this study, whereas the extended score including tendinitis and bursitis specifically addressed the heel. The importance of composite scores in assessing enthesitis can be observed in an ongoing clinical trial of a phosphodiesterase-4 inhibitor in patients with PsA, in which the PsAMRIS composite score was incorporated [11].

The recently published OMERACT heel enthesitis scoring system (HEMRIS) is a promising scoring system for trained MRI readers [12]. HEMRIS includes inflammatory findings and structural MRI findings for the assessment of heel enthesitis, and the final score depends on a consensus-based approach of MR images by the investigators. Most of the parameters, except tendon thickening and bone spur, scored in HEMRIS were evaluated in ACHILLES as well. The factor that differentiates ACHILLES from HEMRIS is the scoring of inflammatory and structural changes. HEMRIS, in particular, is a semi-quantitative scale of 0–3 (none/mild/moderate/severe) to score inflammatory and structural pathologies which are summed up to provide the total entheseal inflammation score and the total entheseal structural damage score, respectively.

In ACHILLES, a comprehensive evaluation of various inflammatory and structural pathologies was performed. The MRI evaluations at screening, Week 24 and Week 52, in combination with clinical assessments, are expected to shed light on improving imaging endpoints to assess and monitor enthesitis.

### Limitations

This study has a few limitations to be considered. No contrast agent was used and, in some cases, that may hamper the evaluation of inflammation. The original PsAMRIS categorised BME quantification as 0 to 3 based on the **area** of bone being oedematous, whereas in the modified system, category 1 (1–33%) was subdivided into 1a and 1b based on oedema **length** ≤/> 0.5 cm. Therefore, it is not possible to calculate a mean score based on the modified PsAMRIS.

In addition, the introduced sub-categories resulted in an uneven distribution of the BME quantification categories; 0–1–2-3 in the original PsAMRIS compared to 0-1a-1b-2-3 in the modified system adapted to the heel. Nevertheless, the sub-categories may allow for greater sensitivity when evaluating at different time points for improvement or worsening of BME based on shift analyses.

Another limitation is the PsAMRIS for bone erosion. If PsAMRIS is employed for the calcaneus, due to its large structure than the carpal bones, it is unlikely that bone erosions in PsAMRIS categories higher than 3 (21–30% of bone eroded) can be observed. This may result in a reduced discriminating power of this score when applied to the heel. Further, MRI reading was hampered in some cases by not being able to measure the erosions as the ‘healthy’ border of the bone was missing due to erosions by the time the first MRI in the study was performed. In the absence of standardised measuring tools for quantifying bone erosion and bone oedema, the quantifications were performed based on the readers’ estimation that could also pose to be a limitation.

## Conclusion

ACHILLES is the largest trial in patients with SpA with clinical and MRI-positive heel enthesitis followed up to 52 weeks. A modified PsAMRIS was applied for the quantification of bone oedema and location of bone erosion. All heel MRIs were evaluated for entheseal changes based on tendinitis, bone oedema, bone erosion, bursitis and periarticular inflammation. Analyses of ACHILLES imaging data at screening, Week 24 and Week 52, in combination with clinical data, will provide deeper insights into the diagnostic challenges of enthesitis. The adaptations of the PsAMRIS introduced in the ACHILLES study represent a new attempt at developing a specific, sensitive and reliable imaging measure for the evaluation of enthesitis in SpA.

## Supplementary Information

**Additional file 1.** Study design.

**Additional file 2.** MRI sequence parameters.

## Data Availability

The datasets generated during and/or analysed at the end of the current study are not publicly available. Novartis is committed to sharing with qualified external researchers’ access to patient level data and supporting clinical documents from eligible studies. These requests are reviewed and approved on the basis of scientific merit. All data provided is anonymized to respect the privacy of patients who have participated in the trial in line with applicable laws and regulations. The data may be requested from the corresponding author of the manuscript.

## Abbreviations

axSpA: Axial spondyloarthritis
BME: Bone marrow oedema
LEI: Leeds Enthesitis Index
MASES: Maastricht Ankylosing Spondylitis Enthesitis Score
MRI: Magnetic resonance imaging
OMERACT: Outcome Measures in Rheumatology Clinical Trials
PsA: Psoriatic arthritis
PsAMRIS: Psoriatic arthritis magnetic resonance imaging scoring system
SpA: Spondyloarthritis
SPARCC: Spondyloarthritis Research Consortium of Canada
STIR: Short inversion time inversion-recovery
