# Supplementary material for: Achilles tendon enthesitis evaluated by MRI assessments in patients with axial spondyloarthritis and psoriatic arthritis: a report of the methodology of the ACHILLES trial
Source: BMC Musculoskelet Disord. 2020 Nov 21;21:767. doi: 10.1186/s12891-020-03775-4 (PMC7680600; doi:10.1186/s12891-020-03775-4)
Supplement: Supplementary file 1 — Additional file 1. Study design. [file 12891_2020_3775_MOESM1_ESM.docx]

**Additional file 1 Study design**


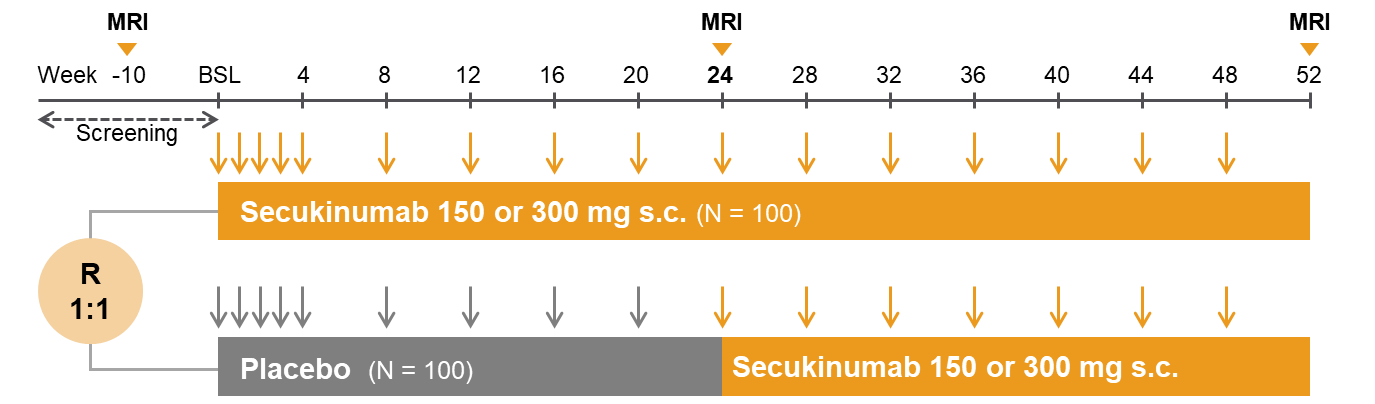


BSL, baseline; MRI, magnetic resonance imaging; R, randomisation; s.c., subcutaneous
