# Supplementary material for: Achilles tendon enthesitis evaluated by MRI assessments in patients with axial spondyloarthritis and psoriatic arthritis: a report of the methodology of the ACHILLES trial
Source: BMC Musculoskelet Disord. 2020 Nov 21;21:767. doi: 10.1186/s12891-020-03775-4 (PMC7680600; doi:10.1186/s12891-020-03775-4)
Supplement: Supplementary file 2 — Additional file 2. MRI sequence parameters. [file 12891_2020_3775_MOESM2_ESM.docx]

**Additional file 2 MRI sequence parameters**

|  | Parameter Ranges | T1 (Sagittal and Transversal) | STIR (Sagittal and Transversal) |
| --- | --- | --- | --- |
| Mandatory settings | Sequence type | TSE/FSE | STIR^a^ |
|  | Dimension | 2D | 2D |
|  | Repetition time - TR (ms) | 400-600 | 2800-3500 |
|  | Echo time - TE (ms) | Maximum 25 | 60-100 |
|  | Inversion time - TI (ms) | N/A | 140-170 |
|  | Slice thickness (mm) | 3 | 3-4 |
|  | Distance factor/gap (mm) | Maximum 10% | Maximum 10% |
|  | In-plane resolution better than (mm x mm) | 0.6 x 0.6 | 0.7 x 0.7 |
|  | Specific settings | - Interpolation off - Do not use parallel acquisition - Adapt the number of slices (approximately 20) so as to cover the complete bone structure by the slice stack | |

^a^Turbo Inversion Recovery, Fast Inversion Recovery, Turbo Inversion Recovery Magnitude or equivalent sequence may be used.

FSE, fast spin-echo; MRI, magnetic resonance imaging; N/A, not applicable; STIR, short inversion time inversion-recovery; TSE, turbo spin-echo
